# Endothelial NOX5 overexpression induces changes in the cardiac gene profile: potential impact in myocardial infarction?

**DOI:** 10.1007/s13105-023-00975-z

**Published:** 2023-08-11

**Authors:** Adriana Cortés, Javier Marqués, Álvaro Pejenaute, Elena Ainzúa, Eduardo Ansorena, Gloria Abizanda, Felipe Prósper, Carlos de Miguel, Guillermo Zalba

**Affiliations:** 1 Department of Biochemistry and Genetics, University of Navarra, Pamplona, Spain; 2 Navarra Institute for Health Research (IdiSNA), Pamplona, Spain; 3 Hematology Service, Clínica Universidad de Navarra, University of Navarra, Pamplona, Spain; 4 CIBERONC, Madrid, Spain

**Keywords:** Oxidative stress, NOX5, Myocardial infarction, Echocardiography

## Abstract

Cardiovascular diseases and the ischemic heart disease specifically constitute the main cause of death worldwide. The ischemic heart disease may lead to myocardial infarction, which in turn triggers numerous mechanisms and pathways involved in cardiac repair and remodeling. Our goal in the present study was to characterize the effect of the NADPH oxidase 5 (NOX5) endothelial expression in healthy and infarcted knock-in mice on diverse signaling pathways. The mechanisms studied in the heart of mice were the redox pathway, metalloproteinases and collagen pathway, signaling factors such as NFκB, AKT or Bcl-2, and adhesion molecules among others. Recent studies support that NOX5 expression in animal models can modify the environment and predisposes organ response to harmful stimuli prior to pathological processes. We found many alterations in the mRNA expression of components involved in cardiac fibrosis as collagen type I or TGF-β and in key players of cardiac apoptosis such as AKT, Bcl-2, or p53. In the heart of NOX5-expressing mice after chronic myocardial infarction, gene alterations were predominant in the redox pathway (NOX2, NOX4, p22phox, or SOD1), but we also found alterations in VCAM-1 and β-MHC expression. Our results suggest that NOX5 endothelial expression in mice preconditions the heart, and we propose that NOX5 has a cardioprotective role. The correlation studies performed between echocardiographic parameters and cardiac mRNA expression supported NOX5 protective action.

**Supplementary Information:**

The online version contains supplementary material available at 10.1007/s13105-023-00975-z.

## Introduction

Cardiovascular diseases (CVDs) constitute the main cause of morbimortality worldwide, and the ischemic heart disease is the greatest threat to life. Several risk factors such as high cholesterol, hypertension, diabetes, obesity, sedentary lifestyle, poor eating habits, alcoholism, smoking, and stress derive to ischemic heart disease. The decrease in cardiac perfusion and, subsequently, ischemic heart disease can lead to myocardial infarction that consists in the death of cardiac tissue inducing myocardial necrosis [1]. Once the heart is harmed, it triggers numerous mechanisms and pathways involved in cardiac repair and postinfarction remodeling. The healing of the infarcted myocardium starts with an intense inflammatory response in which inflammatory leukocytes clean the wound from dead cells. Then, it follows the proliferative state that is characterized by the suppression of proinflammatory signaling and infiltration of the infarct with mesenchymal cells that secrete matrix proteins. Finally, the maturation phase related to quiescence, removal of the reparative cells, and cross-linking of the matrix to form the collagen scar takes place [8].

Oxidative stress constitutes one of the most relevant molecular mechanisms underlying CVDs and is produced by a pathological accumulation of reactive oxygen species (ROS) and the antioxidant system failure. These ROS participate in several processes leading to heart degeneration as it negatively affects myocardial calcium (Ca^2+^) handling and contributes to cardiac remodeling by inducing hypertrophic signaling, apoptosis, and necrosis [24].

The family of the NADPH oxidases (NOXs) are one of the main sources of ROS in the cardiovascular system and are widely expressed among the different cell types such as endothelial cells, smooth muscle cells, adventitial cells, and cardiomyocytes. The NOX family is composed by five homologous (NOX1-5) and two dual oxidases (DUOX1/2) [27]. NOX2 and NOX4 are the most expressed homologs in the heart while NOX5 is mostly expressed in endothelial cells and vascular smooth muscle cells [29]. The NOX family has emerged as the primary oxidase system underlying oxidative stress in vascular diseases including myocardial infarction. In this way, NOX2 and p22phox, which is a regulatory subunit of many NOX isoforms (NOX1-4), are increased in mice hearts after myocardial infarction [9]. At the same time, NOX2 contributes to adverse left ventricular remodeling and contractile dysfunction in infarcted mice [20] while its expression increased in human hearts after myocardial infarction [16].

The NOX5 homolog has been studied in a less extent because of its evolutionary loss in the rodent genome, and its relevance in heart pathophysiology remains unclear. However, it has been described that NOX5 expression increased in human intramyocardial blood vessels and cardiomyocytes after myocardial infarction [12]. The aim of the present investigation was to determine whether NOX5 endothelial expression in mice could alter cardiac gene expression prior to a chronic infarction and its influence in the heart response to the ischemic event.

## Materials and methods

### Conditional knock-in infarcted mouse model

C57BL/6 conditional knock-in mice expressed NOX5-β (the predominant NOX5 isoform in human vasculature) only in endothelial cells after induction with tamoxifen (40 mg/kg) by intraperitoneal injection on 3 non-consecutive days (NOX5^+/−^CRE^+/−^). This, activated the CRE recombinase, expressed only in endothelial cells under *Cdh5* promoter, triggering the transgenesis. The endothelial cell-specific CRE recombinase-expressing mice (CRE^+/−^), gently provided by Wang et al. [33], was used as control mice (*Cdh5*(PAC)-CreERT2; CRE^+/**−**^).

Chronic myocardial infarction was performed in adult mice (15-week-old, male, *n* = 16/group) by permanent ligation of the left anterior descending coronary artery (LAD) as previously described [21]. Briefly, mice were anesthetized, intubated, and mechanically ventilated using a small animal respirator. After waxing and disinfection of the area (left chest), a left lateral thoracotomy (at the 4th intercostal space) was performed. After opening the pericardium, the LAD is visualized, and permanent ligation is carried out with a Prolene 7/0 (W8702, ETHICON), a nylon surgical non-resorbable suture. The incision was closed 15 min after causing the infarction after checking the absence of hemorrhages. Once the recovery and stabilization of mice spontaneous respiration were verified, the endotracheal tube was removed, and mice were kept in a cage warmed with a thermal blanket until animals fully recovered from general anesthesia. The days after surgery, mice recovery was evaluated, and weight was recorded every 2 days. To determine the cardiac effect of NOX5 endothelial expression in mice at baseline, we used animals (12-week-old, male, *n* = 9/group) with no myocardial infarction and referred as “healthy” CRE^+/−^ and NOX5^+/−^CRE^+/−^ mice. We have previously reported that overexpression of NOX5 in endothelial cells was associated with increased ROS production [6].

Experiments were performed in accordance with European Community Council Directives (2010/63/EU) guidelines for the care and use of laboratory animals and were approved by the University of Navarra Animal Research Review Committee (Protocol 106-17).

### Echocardiography data

The echocardiography parameters analyzed in the present study were obtained using Vevo 770 high-resolution ultrasound system (Visual Sonics Inc., Toronto, Canada) as previously described [21]. Briefly, mice chest was waxed and they were anesthetized with 2–3% isoflurane (IsoVet, B. Braun VetCare S.A., Tuttinglen, Germany) maintained until data recording was completed. Mice were placed on a heating pad to maintain corporal heat, and heart rate was monitored during echocardiography evaluation. Before the infarcted mice group were euthanized, three measurements were performed: 3 days before surgery (basal echocardiography), 2 and 28 days after LAD ligation. In the present study, we analyzed the measurements obtained after LAD ligation using the Simpson’s two-dimensional method. This method is the one recommended to analyze the left ventricular ejection fraction, and the parameters obtained are as follows: left ventricular volume in diastole (Simpson Vold), left ventricular volume in systole (Simpson Vols), and left ventricular ejection fraction (Simpson EF). No echocardiography studies were performed in the healthy mice.

### Cardiac tissue processing: RNA extraction and cDNA generation

After the sacrifice of animals, hearts were extracted and cut in 0.1 M phosphate-buffered saline (PBS) (pH = 7.4) separating the apical part of the organ. The apical section was stored for histological procedures, and the rest of the heart was used for gene and protein expression analysis. In the present study, we only analyzed the mRNA expression. For this purpose, heart samples were homogenized in 1 mL of Trizol (Thermo Fisher, Waltham, Massachusetts) using an ULTRA-TURRAX T25 Homogenizer (IKA, Staufen, Germany), and the standard protocol was followed. Total RNA concentration and purity were determined using a Nanodrop ND-1000 spectrophotometer (Thermo Scientific, Rockford, IL, USA). After RNA samples extraction, cDNA was obtained from 1.5 µg of RNA by reverse transcription with SuperScript III cDNA Synthesis Kit (Thermo Scientific, Rockford, IL, USA).

### Quantitative real-time PCR assay

Real-time PCRs were performed using Master Mix iQ™ SYBR® Green Supermix (Bio-Rad, Hercules, California) in an CFX384 Touch real-time PCR Detection System (Bio-Rad, Hercules, CA, USA). In these experiments, standard PCR protocol was followed: 15 min incubation at 95 °C (ensuring enzyme activation), 20 s at 95 °C, 15 s (annealing at the specific melting temperature of each gene) and 10 s of elongation at 72 °C (the last three steps were repeated for 40 times) to obtain the threshold cycle (C_T_). 2^−∆∆CT^ (∆∆C_T_ = (C_T_ analyzed gene – C_T_ control gene) - ∆C_T_ control gene mean) method was introduced for calculation of the relative gene expression with glyceraldehyde 3-phosphate dehydrogenase (GAPDH) as the loading control. Finally, they were normalized with the CRE^+/−^ control mice. Primers used for cDNA amplification are listed in Table S1.

### Western blotting assay

The whole hearts extracted from mice were mechanically triturated and sonicated in RIPA Buffer (1% NP-40, 150 mM NaCl, 50 mM Tris pH = 8.1, 0.1% SCS and 0.5% sodic dexycholate). For each assay, 30 µg of protein were loaded in 10% acrylamide gels and underwent electrophoresis for 1 h and 45 min at 130 V. After that, proteins were transferred from the gels to nitrocellulose membranes (GE10600003, Merck KGaA®) at 350 mA for 1 h. Then, membranes were blocked in 5% BSA 0.05% Tween® TBS for 1 h. Primary antibodies were diluted 1:1000 in the blocking solution and incubated for 16 h at 4 °C. The primary antibodies used were purchased from Cell SignalingTM: JNK Antibody #9252 Phospho-SAPK/JNK (Thr183/Tyr185) (Ref: #9251), p38 MAPK Antibody (Ref: #9212), Phospho-p38 MAPK (Thr180/Tyr182) (Ref: #9211), p44/42 MAPK (Erk1/2) (Ref: #9102), Phospho-p44/42 MAPK (Erk1/2) (Thr202/Tyr204) (Ref: #9101). Membranes were washed three times with 0.05% Tween® TBS for 10 min, incubated with the pertinent mouse (NA931V, GE Healthcare, Merck KGaA®) or rabbit (NA934V, GE Healthcare, Merck KGaA®) antibodies for 1 h at room temperature, and washed finally three more times. Blots were quantified using Image-J™ software (v1.53), and phosphorylation rate was calculated. Membranes were cropped to ease figure understanding.

### Statistical analysis

Data show the mean ± standard error of the mean (SEM). The significance was calculated using a *t*-test when data presented a normal distribution (Shapiro–Wilk and Levene test). When data did not present a normal distribution, the significance was estimated using the Mann-Whitney test; in these cases, the nonparametric test was indicated in the pertinent figure legends. The statistical analysis was performed using GraphPad Prism 8 (GraphPad®, San Diego, CA). The bivariate correlations between echocardiography parameters and gene expression of the infarcted mice were obtained using the program the statistical package IBM SPSS Statistics software (SPSS). Statistical significance was established as *p* < 0.05.

## Results

### Cardiac mRNA expression at baseline in the knock-in mice

**Fig. 1:**
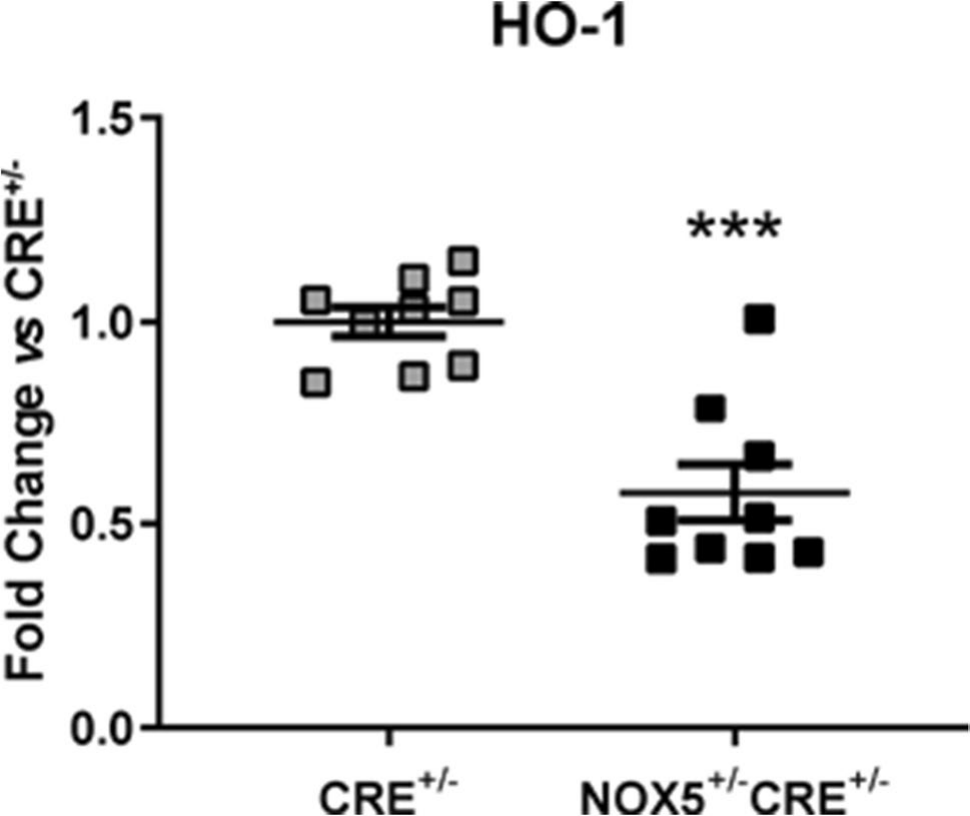
mRNA expression of HO-1 in the heart of healthy mice. HO-1 mRNA expression in the NOX5-expressing mice (NOX5^+/−^CRE^+/−^) and the control mice (CRE^+/−^) at baseline. ***p < 0.001 vs control CRE^+/−^ mice. Mann-Whitney test was used to analyze HO-1 data. Results expressed as mean ± SEM. CRE^+/−^; *n* = 9, NOX5^+/*−*^CRE^+/*−*^; *n* = 9

Healthy NOX5^+/−^CRE^+/−^mice presented no differences in the mRNA levels of most of the redox pathway components analyzed including NOX2, NOX4, p22phox, the superoxide dismutase enzymes (SOD1-3), catalase nor in the endothelial nitric oxide synthase (eNOS) compared with controls (Fig. S1). In contrast, hemoxigenase 1 (HO-1) cardiac mRNA levels decreased in the healthy NOX5^+/−^CRE^+/−^ mice compared to healthy CRE^+/−^ mice (Fig. 1).

**Fig. 2:**
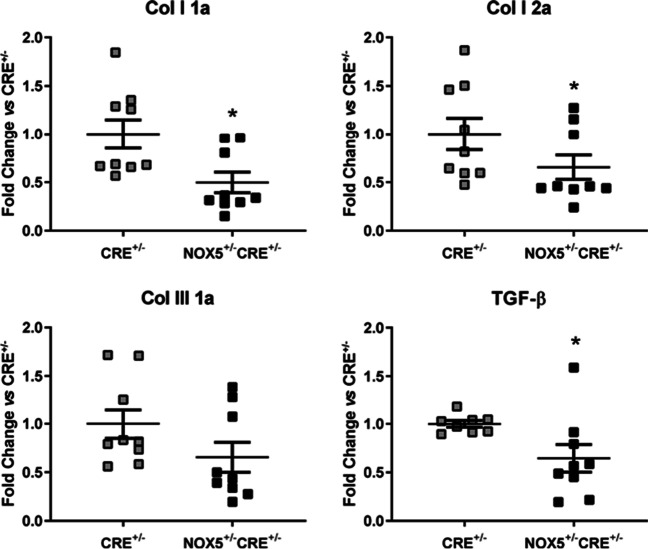
mRNA expression of fibrotic components in the heart of healthy mice. Col I 1a, Col I 2a, Col III 1a, and TGF-β mRNA expression in the NOX5-expressing mice (NOX5^+/*−*^CRE^+/−^) and the control mice (CRE^+/−^) at baseline. *p < 0.05 vs control CRE^+/−^ mice. Mann-Whitney test was used to analyze Col I 1a, Col 1 2a, and Col III 1a data. Results expressed as mean ± SEM. CRE^+/−^; *n* = 9, NOX5^+/−^CRE^+/−^; *n* = 9

Concerning to the molecular mechanisms involved in cardiac remodeling and in cardiac fibrosis specifically, we studied the effect of NOX5 expression per se in the heart of healthy NOX5^+/−^CRE^+/−^ mice. First, we found decreased mRNA levels in the two components of the collagen type I, which predominate in heart tissue known as collagen type I 1α and collagen type I 2α (Col I 1a and Col I 2a, respectively) compared to healthy CRE^+/−^ mice (Fig. 2). No differences were found in the second predominant collagen in the heart known as collagen type III 1α (Col III 1a) although its levels tended to be lower in the healthy NOX5^+/−^CRE^+/−^ mice compared to healthy CRE^+/−^ mice. The expression of the transforming growth factor β (TGF-β) in the heart of healthy mice also decreased in NOX5^+/−^CRE^+/−^ mice compared to healthy CRE^+/−^ mice (Fig. 2).

NOX5 endothelial expression induced no modifications in the collagen IV 1α (Col IV 1a), connective tissue growth factor (CTGF) nor in the fibronectin. Additionally, NOX5 did not affect extracellular matrix homeostasis as no differences were found in metalloproteinases (MMP2, MMP9, or MMP10) or in its inhibitor (metalloproteinase inhibitor 2; TIMP2) between groups (Fig. S2).

**Fig. 3:**
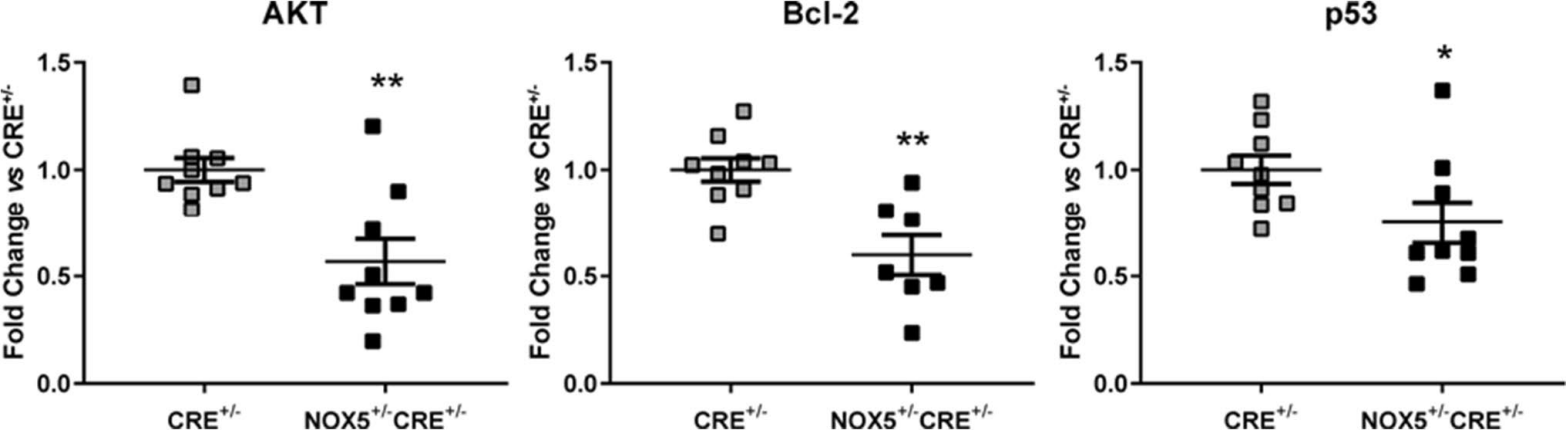
mRNA expression of AKT, Bcl-2 and p53 in the heart of healthy mice. AKT, Bcl-2, and p53 and mRNA expression in the NOX5-expressing mice (NOX5^+/−^CRE^+/−^) and the control mice (CRE^+/−^) at baseline. *p < 0.05, **p < 0.01 vs control CRE^+/−^ mice. Mann-Whitney test was used to analyze AKT data. Results expressed as mean ± SEM. CRE^+/−^; *n* = 9, NOX5^+/−^CRE^+/−^; *n* = 9

Additionally, cardiac remodeling involves mechanisms related to the control of the cell cycle triggering cardiac tissue proliferation and apoptosis. In the heart of the healthy NOX5^+/−^CRE^+/−^ mice, AKT, Bcl-2, and p53 mRNA levels decreased compared to healthy CRE^+/−^ mice (Fig. 3).

**Fig. 4:**
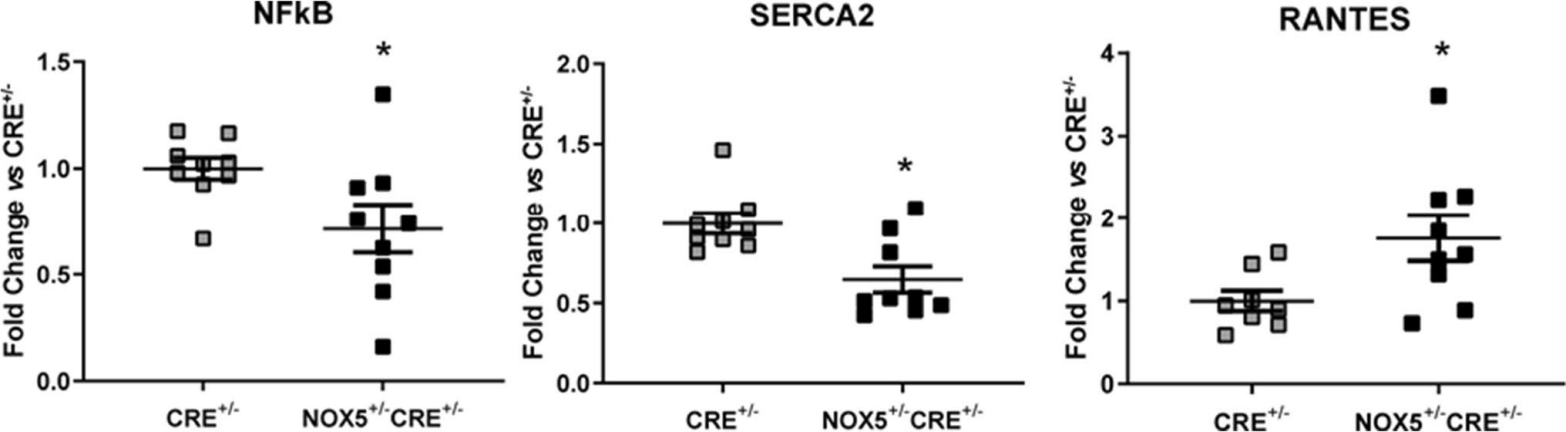
mRNA expression of the inflammatory pathway components in the heart of healthy mice. NFκB, SERCA2, and RANTES mRNA expression in the NOX5-expressing mice (NOX5^+/−^CRE^+/−^) and the control mice (CRE^+/−^) at baseline. *p < 0.05 vs control CRE^+/−^ mice. Results expressed as mean ± SEM. CRE^+/−^; *n* = 9, NOX5^+/−^CRE^+/−^; *n* = 9

Then, we studied the inflammatory pathway component expression. In this way, it has been previously reported that NOX5 endothelial expression induces cPLA_2_ overexpression in the heart of healthy NOX5^+/−^CRE^+/−^ mice compared to healthy CRE^+/−^ mice [21] suggesting that the prostaglandin inflammatory pathway could be altered in these animals. In the present study, cardiac mRNA levels of the nuclear factor kappa light chain enhancer of activated B cells (NFκB) and the sarco/endoplasmatic reticulum Ca^2+^-ATPase 2 (SERCA2) decreased in the healthy NOX5^+/−^CRE^+/−^ mice compared to healthy CRE^+/−^ mice, whereas the chemokine regulated upon activation normal T-cell expressed and secreted (RANTES) increased (Fig. 4). No differences were found between groups in the expression of cardiotrophin 1 (CTF1), interleukin 6 (IL-6), nor in tumor necrosis factor α (TNF-α) mRNA levels compared to control CRE^+/−^ mice although the last two tended to be lower in the NOX5^+/−^CRE^+/−^ mice (Fig. S3).

**Fig. 5:**
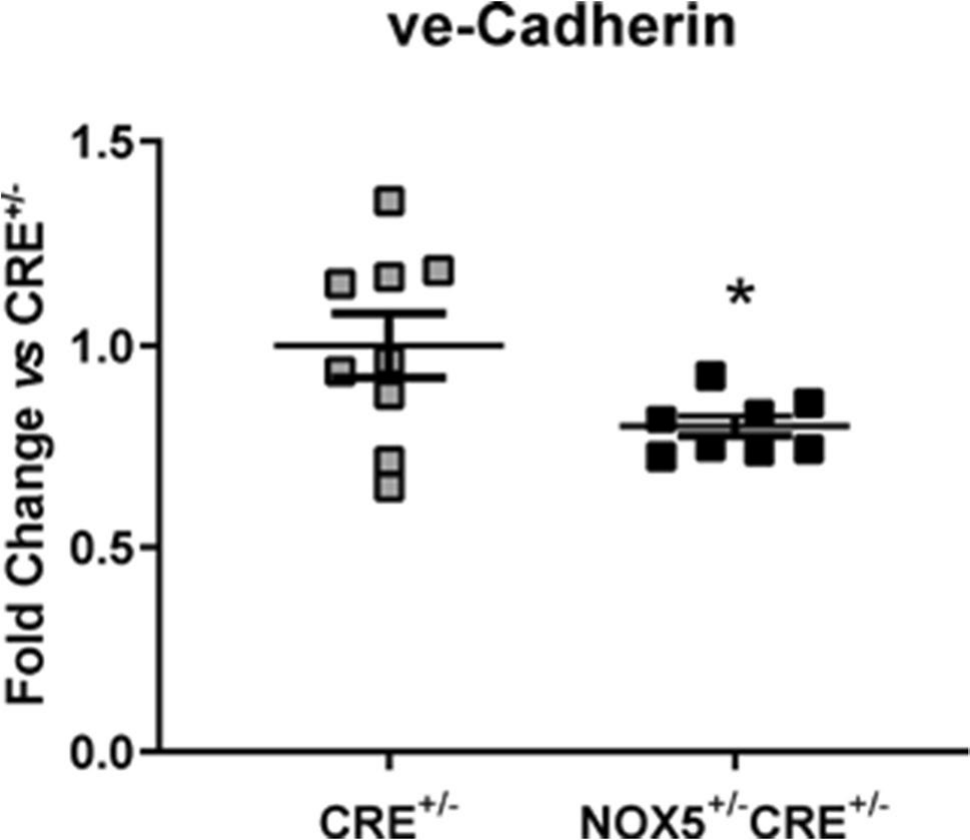
mRNA expression of ve-Cadherin in the heart of healthy mice. ve-Cadherin mRNA expression in the NOX5-expressing mice (NOX5^+/−^CRE^+/−^) and the control mice (CRE^+/−^) at baseline. *p < 0.05 vs control CRE^+/−^ mice. Results expressed as mean ± SEM. CRE^+/−^; *n* = 9, NOX5^+/−^CRE^+/−^; *n* = 9

Finally, the vascular endothelial (ve)-cadherin mRNA levels decreased in the heart of healthy NOX5^+/−^CRE^+/−^ mice compared to healthy CRE^+/−^ mice (Fig. 5). No differences were found in the vascular cell adhesion molecule-1 (VCAM-1), in the intercellular adhesion molecule-1 (ICAM-1), or in the two myosin heavy chain molecules (α-MHC and β-MHC) analyzed (Fig. S4).

### Cardiac protein expression at baseline in the knock-in mice

NOX5^+/−^CRE^+/−^ mice exhibited a 10-fold increase in the rate of ERK phosphorylation compared to CRE^+/−^ mice, although we did not observe significant differences (Fig. S5). There were no differences between NOX5^+/−^CRE^+/−^ and CRE^+/−^ mice in the rate of phosphorylation of p38. Finally, there were no differences between NOX5^+/−^CRE^+/−^ and CRE^+/−^ mice in the rate of phosphorylation of JNK.

### Echocardiography data

In previous studies, we did not identify any difference between NOX5^+/−^CRE^+/−^ and control CRE^+/−^ mice in the echocardiography parameters analyzed. The parameters analyzed had been as follows: the mitral valve wave E (MV_E), the mitral valve wave A (MV_A), the mitral valve waves ratio E/A (MV_E/A), the E′, the A′, the E′/A′, the isovolumetric contraction time (IVCT), the isovolumetric relaxation time (IVRT), a, b, the index tei, the E/E′, the interventricular septum (IVS), the left ventricular internal diameter (LVID), the left the ventricular posterior wall (LVPW), the left ventricle volume (LV Vol), the ejection fraction (EF), the fractional shortening (FS), and the left ventricle mass (LV Mass) [21]. In the present study, no differences were found in any of the three Simpson’s echocardiography parameters analyzed (Simpson Volume diastole, Simpson Volume systole or Simpson Ejection Fraction) (Table 1).

**Table 1:** Echocardiographic Simpson parameters in infarcted mice 2 days and 28 days after LAD ligation

	2 days		28 days	
	CRE^+/−^	NOX5^+/−^CRE^+/−^	CRE^+/−^	NOX5^+/−^CRE^+/−^
Simpson volume diastole (µL)	88.232 ± 2.763	89.107 ± 3.643	159.094 ± 27.635	128.403 ± 10.204
Simpson volume systole (µL)	55.825 ± 2.436	57.399 ± 3.266	86.832 ± 12.733	82.251 ± 10.169
Simpson ejection fraction (%)	36.638 ± 1.950	36.057 ± 1.676	36.925 ± 2.833	38.205 ± 3.029

Data are represented as the mean ± SEM. CRE^+/**−**^, mice with the CRE genotype (*n* = 12); NOX5^+/**−**^CRE^+/**−**^, mice with the NOX5^+/**−**^CRE^+/**−**^ genotype (*n* = 13)

### Cardiac mRNA expression in mice after chronic myocardial infarction

**Fig. 6:**
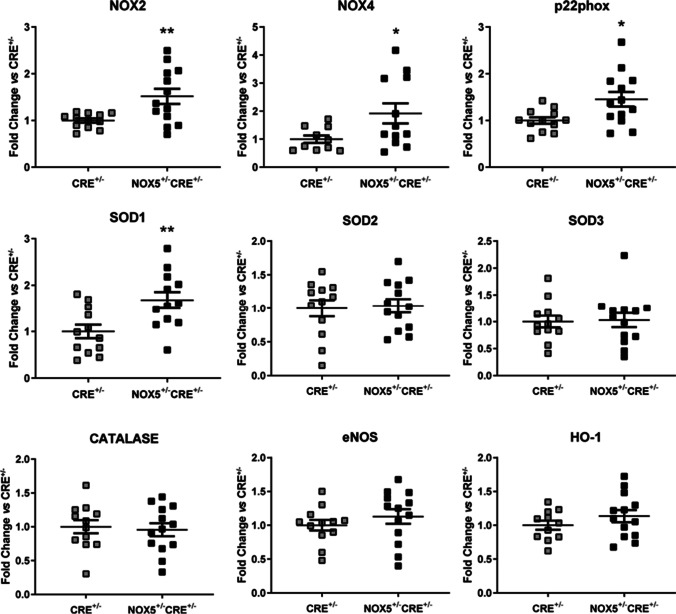
mRNA expression of redox pathway components in the heart of infarcted mice. NOX2, NOX4, p22phox, SOD1, SOD2, SOD3, Catalase, eNOS, and HO-1 mRNA expression in the NOX5-expressing infarcted mice (NOX5^+/−^CRE^+/−^) and the control infarcted mice (CRE^+/−^). *p < 0.05, **p < 0.01 vs control CRE^+/−^ mice. Results expressed as mean ± SEM. CRE^+/−^; *n* = 12, NOX5^+/−^CRE^+/−^; *n* = 13

The heart of the infarcted NOX5^+/−^CRE^+/−^mice presented significative differences in the mRNA levels of the redox pathway compared to the control group. NOX2, NOX4, p22phox, and SOD1 were upregulated in the NOX5^+/−^CRE^+/−^ mice compared to CRE^+/−^ mice (Fig. 6).

No differences between groups were observed in the agents involved in cardiac fibrosis or in the extracellular matrix mediators as the different collagen types, the CTGF, the fibronectin, the TGF-β, and in the metalloproteinases pathway components mRNA levels (Fig. S6). Additionally, no significative modifications in the infarcted mice mRNA levels of AKT, Bcl-2, nor in p53 were detected compared to CRE^+/−^ mice (Fig. S7). Identical result was obtained when the inflammatory agents were analyzed (Fig. S8). In the infarcted mice, the tumor necrosis factor β (TNF-β), the atrial natriuretic factor (ANF), and the cerebral natriuretic factor (BNP) gene expression were evaluated and presented no differences between groups (Fig. S8).

**Fig. 7:**
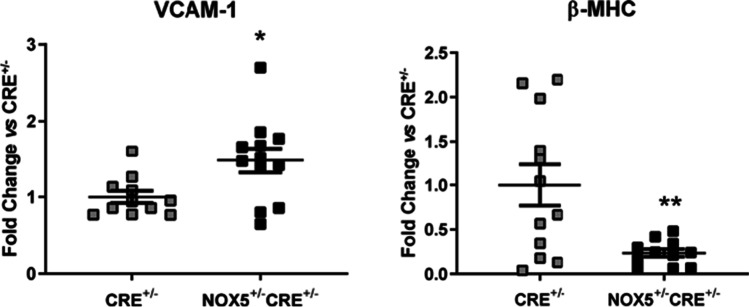
mRNA expression of VCAM-1 and β-MHC in the heart of infarcted mice. VCAM-1 and β-MHC mRNA expression in the NOX5-expressing infarcted mice (NOX5^+/−^CRE^+/−^) and the control infarcted mice (CRE^+/−^). *p < 0.05, **p < 0.01 vs control CRE^+/−^ mice. Mann-Whitney test was used to analyze VCAM-1 data. Results expressed as mean ± SEM. CRE^+/−^; *n* = 12, NOX5^+/−^CRE^+/−^; *n* = 13

Finally, NOX5 expression in the infarcted mice induced an increase in VCAM-1 expression compared to CRE^+/−^ mice, while β-MHC mRNA expression was strongly decreased compared to CRE^+/−^ mice (Fig. 7). Concerning to ICAM-1, ve-cadherin, or α-MHC mRNA levels, we detected no differences between groups (Fig. S9).

### Cardiac protein expression in mice after chronic myocardial infarction

Infarction did not induce a significant increase in ERK phosphorylation in either CRE^+/−^ or NOX5^+/−^CRE^+/−^ mice (Fig. S10). Similarly, in infarcted mice, there were no differences between NOX5^+/−^CRE^+/−^ and CRE^+/−^ mice in the rate of phosphorylation of p38 nor JNK.

### Bivariate correlations

Correlations were performed between the echocardiographic parameters and the mRNA expression of those genes that resulted in significant alterations (NOX2, NOX4, p22phox, V-CAM1, and β-MHC). These resulted in many significant interactions differentially produced depending on NOX5 expression (Fig. S11–S12).

## Discussion

ROS production and their function as signaling molecules are key in heart development and homeostasis and participate in the pathogenesis of CVDs [24]. The NOX family constitute the main source of ROS in the cardiovascular system and the oxidative stress derived from these enzymes are related to CVDs. Interestingly, several experimental studies support that NOX5 expression may be necessary to attenuate cardiovascular complications in response to harmful stimuli [10, 25]. In the present study, our data allow us to suggest that ROS derived from NOX5 expression preconditionate the heart through gene expression modifications. First of all, mRNA levels of HO-1, a protective molecule that participates in endothelial cells homeostasis, were strongly decreased in our healthy NOX5^+/−^CRE^+/−^ mice. Given that HO-1 may palliate oxidative damage and participate in endothelium repair after brain ischemic injury [7], our finding suggests that NOX5 expression in these mice may be predisposing the heart to vascular damage.

NOX5-activity is regulated by many stimuli, but one of the main regulators is the intracellular Ca^2+^ level [12]. Overexpression of human NOX5 in cardiomyocytes of rats exacerbated cardiac hypertrophy during heart failure, a pathological process that is mediated by intracellular Ca^2+^ level [37]. Nevertheless, our results showed that endothelial NOX5 overexpression did not alter cardiac function even after myocardial infarction. Given that that in our study the overexpression of NOX5 was driven to endothelial cells, the oxidative stress-associated would be limited only to the vascular wall without affecting the cardiomyocytes. SERCA2 is responsible of endoplasmic reticulum Ca^2+^ uptake, and its deficit induces postischemic myocardial relaxation impairment and larger infarct size in mice [30]. Subsequently, alterations in SERCA2 can modify several molecular processes related to Ca^2+^ signaling as the contraction-relaxation process but also regulate NOX5 activity [37]. SERCA2 Ca^2+^ channel expression was downregulated in the heart of the healthy NOX5^+/−^CRE^+/−^ mice suggesting a precondition of the heart that could have worsened its response to chronic infarction. Finally, in the healthy NOX5^+/−^CRE^+/−^ mice, no changes in the mRNA levels of any member of the NOX family nor in most of the antioxidant agents analyzed were found.

The perpetuation of an oxidative stress environment can trigger cardiac remodeling including molecular mechanisms involved in cardiac fibrosis through extracellular matrix modifications. The extracellular matrix is formed by diverse extracellular molecules primarily comprising collagens (Col I–IV) being the Col I and the Col III the most expressed in the heart accounting for approximately 85% and 11% of the total collagen respectively [34]. It also contains metalloproteinases (MMP2, MMP9, MMP10 and TIMP2), fibronectin and CTGF. In cultured human glomerular mesangial cells, NOX5-silencing induced collagen type I and type IV decrease [19, 35]. In addition, a knock-in mouse expressing NOX5 in the vascular smooth muscle cells and in endothelial cells, presented collagens (Col III and Col IV) upregulation in renal tissues [13]. In the present study, we found lower levels of the collagen type I (Col I 1a and Col I 2a) in the heart of healthy NOX5^+/−^CRE^+/−^ mice. Moreover, the cardiac presence of Col III tended to be lower in the healthy the NOX5^+/−^CRE^+/−^ mice suggesting collagen production attenuation in these mice. However, this downregulation of collagen production apparently has no effect in the phenotype of cardiac tissue as we previously published no differences in cardiac fibrosis after myocardial infarction [21]. One of the most studied mediators in cardiac fibrosis is TGF–β, which is involved in cardiac remodeling, probably mediated by ROS. Oxidative stress stimulate TGF–β through the activation of NFκB [26]. Interestingly, NOX5 is known to be induced by TGF–β in human hepatic stellate cells and to increase Col I production in these cells [2]. Our results showed lower cardiac expression of TGF-β in healthy NOX5^+/−^CRE^+/−^ mice comparing to control mice.

AKT serine/threonine protein kinases are key controllers of the balance between cell survival and apoptosis. They protect cardiomyocytes from apoptosis by inactivating caspases [36]. Interestingly, NOX5 knockdown has been associated with lower phosphorylation levels of AKT in cancer favoring cell survival [3]. In addition, Bcl-2 protect myocardial cells from various stresses and is a key regulator of the mitochondrial pathway of apoptosis in the heart [11]. On the other hand, Bcl-2 blocks p53-mediated apoptosis in cardiac myocytes [14]. p53 is a proapoptotic transcription factor which can be activated by oxidative stress and participates in cardiac homeostasis [22]. Here, we found lower levels of AKT and Bcl-2 in the heart of healthy NOX5^+/−^CRE^+/−^mice suggesting antiapoptotic mechanisms attenuation. However, we also found lower levels of the proapoptotic factor p53. We could not discard that apoptosis in the heart ultimately occurs due to the imbalance between cardiac proapoptotic agents such as p53 and antiapoptotic cardiac agents such as AKT and Bcl-2.

Inflammatory system activation constitutes one of the main cardiac remodeling markers. We previously demonstrated that cPLA_2_ expression is increased in the heart of the healthy NOX5^+/−^CRE^+/−^ mice suggesting an upregulation of the inflammatory prostaglandin pathway in our knock-in mice [21]. Moreover, ROS interfere in cardiac repair by activating NFκB, a key regulator of many genes related to inflammation, cell adhesion, survival, and growth control, triggering the proinflammatory response and promoting fibrogenic cytokine production [17]. Our results showed decreased NFκB mRNA expression levels in healthy NOX5^+/−^CRE^+/−^ mice, although further analysis would be needed to evaluate its activity in order to probe NFκB-pathway regulation. On the other hand, RANTES is an inflammatory chemokine produced by many cells like macrophages, fibroblasts, and endothelial cells. It has unique roles in driving the recruitment of leukocytes, angiogenesis, and fibrosis in various models of chronic inflammation and can activate NFκB pathway [18]. We found that NOX5 endothelial expression induced RANTES upregulation in the heart of healthy mice. These findings together with the decrease in the expression of the ve-cadherin (an indicator of vascular integrity) in the healthy NOX5^+/−^CRE^+/−^ mice suggest that NOX5 could be altering vascular homeostasis and influencing in heart response to damage.

We did not perform any echocardiography analysis in healthy mice so no correlations test could be done with data obtained from these groups. However, our results suggest that NOX5 expressed at baseline attenuated the expression of genes related with cardiac fibrosis, inflammation, or apoptosis, which could worsen heart response to damage. Definitely, endothelial NOX5 expressed in healthy mice appear to precondition cardiac tissue. This could trigger effects in the pathophysiology of the heart depending on the chronicity of the oxidative stress, or the interaction with more aggressive insults such as hypertension, hypercholesterolemia, coagulation/thrombosis alterations, and endothelial dysfunction.

After myocardial infarction, the heart overproduces ROS as well as antioxidant deficit and subsequently promotes an oxidative stress environment [28]. Many studies have also demonstrated that oxidative damage can trigger most of the postinfarction modifications that are thought to contribute to myocardial remodeling including cardiomyocyte apoptosis, proinflammatory cytokine release, fibrogenesis, cell proliferation, and cardiac hypertrophy [8]. Following the ischemic event, cardiac structural remodeling is continued by scar generation at the site of infarction as well as adaptative alterations in the non-infarcted myocardium, and this process is accompanied by an oxidative stress environment. In our infarcted mice, NOX5 endothelial expression upregulated NOX2, NOX4, p22phox, and SOD1 expression, which suggests that the oxidase might aggravate oxidative damage. Nevertheless, it has been demonstrated a pivotal role of NOX2-mediated cardiac oxidative stress in ischemic preconditioning [4]. Likewise, overexpression of NOX4 in cardiomyocytes results in improved post-MI survival and remodeling [23]. The adaptative alterations that take place in the infarcted heart include cardiac fibrosis with extracellular matrix modifications [8] and an intense systemic and local inflammatory response. The degree of inflammation is decisive in the maintenance of cardiac function and myocardial remodeling [32]. In a proinflammatory environment after myocardial infarction, there is an increase in the expression of adhesion molecules. In the present study, we found that the mRNA levels of VCAM-1 were increased in the infarcted NOX5^+/−^CRE^+/−^ mice. This could be favoring leukocyte recruitment and, subsequently, the inflammatory response. Cardiac muscle contraction is mediated by two molecular motors: α-MHC and β-MHC. β-MHC is the most expressed isoform in ventricles trough mice development while α-MHC is expressed transiently during mouse gestation. After the birth of small rodents, α-MHC predominates in the ventricle, whereas in humans, β-MHC levels remain high. Numerous stimuli can shift the MHC composition of the mammalian heart. Diseases like hypothyroidism, pressure overload, and heart failure all result in a lower α-MHC expression and higher β-MHC in mammals [31]. In the present study, we found a significant decrease in the expression of β-MHC in the infarcted NOX5^+/−^CRE^+/−^ mice as a response to the ischemic event. The expression of β-MHC is thought to be disadvantageous to the mice suffering from cardiovascular severe pathologies being considered a maladaptive response [15]. These findings suggest that the endothelial expression of NOX5 in our mice could favor cardiac response to CVDs. At the same time, NOX5 did not affect mice viability after chronic myocardial infarction, and most of the correlations found between the echocardiography parameters and the cardiac gene expression showed a possible cardioprotective role for NOX5. Recently, in agreement with the present study, Casas et al. described in their infarcted knock-in mice, which expressed NOX5 in endothelial and in circulating blood cells under *Tie2* promoter, no differences in the infarct size nor cardiac functionality after LAD ligation [5].

To conclude, NOX5 endothelial expression in healthy mice induce gene expression alterations and modify the cardiac environment prior to damage. The absence of alterations in the gene expression of the NOX5^+/−^CRE^+/−^ infarcted mice suggests that the LAD ligation induces intense changes in the heart (cardiac remodeling), which predominate over the genotype. Additionally, these results demonstrate the complex participation of NOX5 in the adaptation and repair of the heart to the harmful stimuli, in this case, chronic infarction (Fig. S13). However, the measurement of gene expression and its correlation with cardiac parameters constitutes a limitation to be taken into account in its conclusions. Although NOX5 could be a key player in preconditioning the mouse heart prior to chronic infarction, these results should be used as the basis for larger prospective studies.

### Supplementary Information

Below is the link to the electronic supplementary material.

Supplementary file1 (PDF 1021 KB)
