# Supplementary material for: Endothelial NOX5 overexpression induces changes in the cardiac gene profile: potential impact in myocardial infarction?
Source: J Physiol Biochem. 2023 Aug 11;79(4):787–97. doi: 10.1007/s13105-023-00975-z (PMC10635946; doi:10.1007/s13105-023-00975-z)
Supplement: Supplementary file 1 — Supplementary file1 (PDF 1021 KB) [file 13105_2023_975_MOESM1_ESM.pdf]

Endothelial NOX5 overexpression induces changes in the cardiac gene profile. Potential impact in myocardial infarction?

Adriana Cortés[1,2], Javier Marqués[1,2], Álvaro Pejenaute[1,2], Elena Ainzúa[1,2], Eduardo Ansorena[1,2], Gloria Abizanda[2,3], Felipe Prósper[2,3,4], Carlos de Miguel[1,2] and Guillermo Zalba[1,2]*

[1] Department of Biochemistry and Genetics, University of Navarra, Pamplona (Spain); acortes.3@alumni.unav.es (A.C.); jmarquesc@unav.es (J.M.); apejenaute@alumni.unav.es (A.P.); eainzua@unav.es (E.A.P.); eansorena@unav.es (E.A.); cdmiguel@unav.es (C.de M.); gzalba@unav.es (G.Z.)

[2] Navarra Institute for Health Research (IdiSNA), Pamplona (Spain); (J.M.; A.C.; A.P.; E.A.; G.A.; F.P.; C.deM.; G.Z.)

[3] Hematology Service, Clínica Universidad de Navarra, University of Navarra, Pamplona (Spain); gabizanda@unav.es (G.A.); fprosper@unav.es (F.P.)

[4] CIBERONC, Madrid (Spain); (F.P.)

* Correspondence: gzalba@unav.es; Tel.: +34-948-425600

Table S1: Primers used for cDNA amplification.

Gene	Accession Nº		Primers
NOX2	NM_007807.5	forward	5′-ACTCCTTGGGTCAGCACTGG-3′
		reverse	5′-GTTCCTGTCCAGTTGTCTTCG-3′
NOX4	NM_001285833.1	forward	5′-GGAGACTGGACAGAACGATTCC-3′
		reverse	5′-TGTATAACTTAGGGTAATTTCTAGAGTGAATGA-3′
CATALASE	NM_009804.2	forward	5′-GCTGAGAAGCCTAAGAACGCAAT-3′
		reverse	5′-CCCTTCGCAGCCATGTG-3′
eNOS	NM_008713.4	forward	5′-CTGGAGCACCCCACGCT-3′
		reverse	5′-AGCGGTGAGGGTCACACAG-3′
HO-1	NM_010442.2	forward	5′-CAACATTGAGCTGTTTGAGGAG-3′
		reverse	5′-CTCTGACCAAGTGACGCCAT-3′
p22phox	NM_007806	forward	5′-GCCCTCCACTTCCTGTT-3′
		reverse	5′-GCAGATAGATCACACTGGCAAT-3′
SOD1	NM_011434.1	forward	5′-GGACCTCATTTTAATCCTCACTCTAAG-3′
		reverse	5′-GGTCTCCAACATGCCTCTCTTC-3′
SOD2	NM_013671	forward	5'-CACACATTAACGCGCAGATCA-3′
		reverse	5′-GGTGGCGTTGAGATTGTTCA-3'
SOD3	NM_011435.3	forward	5'-ACACCTTAGTTAACCCAGAAATCTTTTC-3′
		reverse	5′-GGGATGGATCTAGAGCATTAAGGA-3'
ve-CADHERIN	NM_009868.4	forward	5′- TCAACGCATCTGTGCCAGAGAT -3′
		reverse	5′- CACGATTTGGTACAAGACAGTG -3′
TNF-α	NM_013693.3	forward	5′- ATGAGCACAGAAAGCATGATCCGCGAC -3′
		reverse	5′- TCACAGAGCAATGACTCCAAAGTAGACCTG -3′
TNF-β	NM_010735.2	forward	5′- CCCATGGCATCCTGAAAC -3′
		reverse	5′- GGAGGCCTGGAATCCAAT -3′
ANF	NM_008725.3	forward	5′-CGTGCCCCGACCCACGCCAGCATGGGCTCC-3′
		reverse	5′-GGCTCCGAGGGCCAGCGAGCAGAGCCCTCA-3′
BNP	NM_008726.5	forward	5′-AAGGGAGAATACGGCATCATTG-3′
		reverse	5′-ACAGCACCTTCAGGAGATCCA-3′
α-MHC	NM_001164171.1	forward	5′-TAAAATTGAGGACGAGCAGGC-3′
		reverse	5′-TCCAGCTCCTCGATGCGT-3′
β-MHC	NM_080728.3	forward	5′-AGCAGCAGTTGGATGAGCGACT-3′
		reverse	5′-CCAGCTCCTCGATGCGTGCC-3′
CTGF	NM_010217.2	forward	5′-GCTGCCTACCGACTGGAAGAC-3′
		reverse	5′-GAACAGGCGCTCCACTCTG-3′
FIBRONECTIN	NM_001276408.1	forward	5′-CCGGTGGCTGTCAGTCAGA-3′
		reverse	5′-CCGTTCCCACTGCTGATTTATC-3′
MMP2	NM_008610.3	forward	5′-TCGCCCATCATCAAGTTCCC-3′
		reverse	5′-CCTTGGGGCAGCCATAGAAA-3′
MMP9	NM_013599.5	forward	5′-CACCACAGCCAACTATGACC-3′
		reverse	5′-GTCTGGACAGAAACCCCACT-3′
MMP10	NM_019471.3	forward	5′-CCTGTGTTGTCTGTCTCTCCAAGA-3′
		reverse	5′-CGTGCTGACTGAATCAAAGGAC-3′
TIMP2	NM_011594.3	forward	5′-GGTACCAGATGGGCTGTGA-3′
		reverse	5′-CATCCAGAGGCACTCATCCG-3′
ICAM-1	NM_010493.3	forward	5′-CTGGGCTTGGAGACTCAGTG-3′
		reverse	5′-TCTCCGGAAACGAATACACG-3′
VCAM-1	NM_011693.3	forward	5′-GCCTCAACGGTACTTTGGAT-3′
		reverse	5′-CTCAAAACTGACAGGCTCCA-3′
IL6	NM_031168.2	forward	5′-CCGGAGAGGAGACTTCACAG-3′
		reverse	5′-GGAAATTGGGGTAGGAAGGA-3′
CTF1	NM_007795.2	forward	5′-CCGCCAGACACACAACCT-3′
		reverse	5′-ACACCGGTAGCCCTGCAT-3′

AKT	NM_001331107.1	forward	5′-CGTCGCCAAGGATGAGGTTG-3′
		reverse	5′-GTCGTGGGTCTGGAATGAGT-3′
Bcl-2	NM_009741.5	forward	5′-AGGCTGGGATGCCTTTGTGG-3′
		reverse	5′-TGTTTGGGGCAGGTTTGTCG-3′
Collagen type I 1a	NM_007742.4	forward	5′-TCTGACTGGAAGAGCGGAGAG-3′
		reverse	5′-AGACGGCTGAGTAGGGAACA-3′
Collagen type I 2a	NM_007743.3	forward	5′-TGGATACGCGGACTCTGTTG-3′
		reverse	5′-CTGGGCCTTTGATACCTGGA-3′
Collagen type III 1a	NM_009930.2	forward	5′-GGGAGGAATGGGTGGCTATC-3′
		reverse	5′-CATTGCAACTCGGTCATTTT-3′
Collagen type IV 1a	NM_009931.2	forward	5′-CTGGAGAAAAGGGCCAGAT-3′
		reverse	5′-TCCTTAACTTGTGCCTGTCC-3′
NF-kB	NM_008689.2	forward	5′-CCTACGGAACTGGGCAAATGT-3′
		reverse	5′-TCCCCTCTGTTTTGGTTGCT-3′
p53	NM_011640.3	forward	5′-ATGCCCATGCTACAGAGGAG-3′
		reverse	5′-AGACTGGCCCTTCTTGGTCT-3′
RANTES	NM_013653.3	forward	5′-TGCTCCAATCTTGCAGTCGT-3′
		reverse	5′-GCGTATACAGGGTCAGAATCAAG-3′
SERCA-2	NM_009722.3	forward	5′-TACTGACCCTGTCCCTGACC-3′
		reverse	5′-CACCACCACTCCCATAGCTT-3′
TGF-β	NM_011577.2	forward	5′-TCAGACATTCGGGAAGCAGT-3′
		reverse	5′-GCCCTGTATTCCGTCTCCTTG-3′
GAPDH	NM_008084.3	forward	5′-ATGACAACTTTGTCAAGCTCATTT-3′
		reverse	5′-GGTCCACCACCCTGTTGCT-3′

NADPH oxidase 2 (NOX2), NADPH oxidase 4 (NOX4), endotelial nitric oxide syntase (eNOS), hemoxigenase 1 (HO-1), superoxide dysmutase 1 (SOD1), superoxide dysmutase 2 (SOD2), superoxide dysmutase 3 (SOD3), vascular endothelial cadherin (ve-Cadherin), tumor necrosis factor α (TNF-α), tumor necrosis factor β (TNF-β), atrial natriuretic factor (ANF), cerebral natriuretic factor (BNP), myosin heavy chain α (α-MHC), myosin heavy chain β (β-MHC), connective tissue growth factor (CTGF), metalloproteinase 2 (MMP2), metalloproteinase 9 (MMP9), metalloproteinase 10 (MMP10), metalloproteinase inhibitor 2 (TIMP2), intercellular adhesion molecule-1 (ICAM-1), vascular cell adhesion molecule-1 (VCAM-1), interleukin 6 (IL6), cardiotrophin 1 (CTF1), nuclear factor kappa light chain enhancer of activated B cells (NFκB), chemokine regulated upon activation normal T-cell expressed and secreted (RANTES), sarco/endoplasmatic reticulum Ca^{2+}-ATPase 2 (SERCA-2), transforming growth factor β (TGF-β), glyceraldehyde 3-phosphate dehydrogenase (GAPDH).

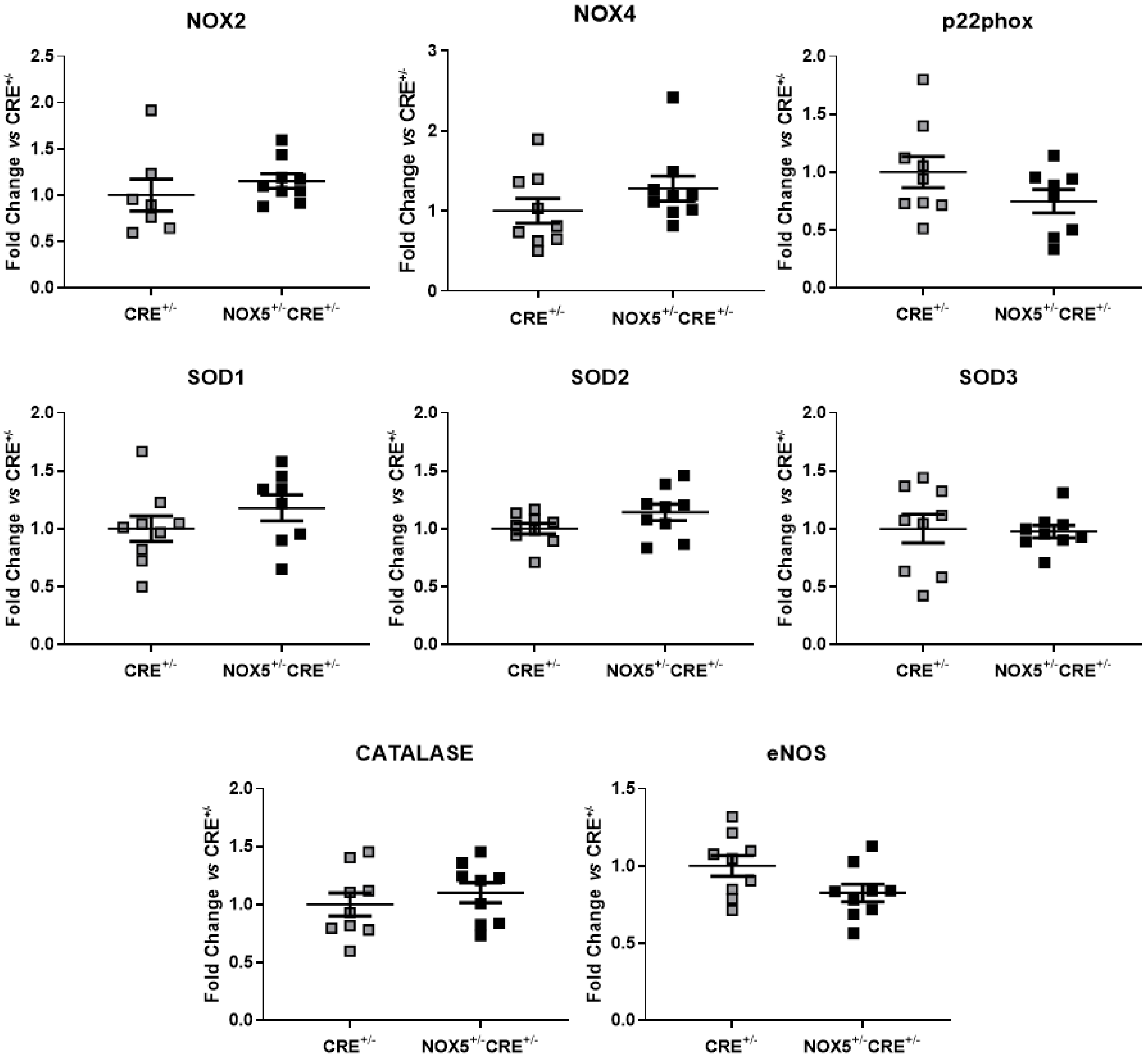

Fig. S1. mRNA expression of redox pathway components in the heart of healthy mice. NOX2, NOX4, p22phox, SOD1, SOD2, SOD3, Catalase and eNOS mRNA expression in the NOX5-expressing mice (NOX5$^{+/-}$CRE$^{+/-}$) and the control mice (CRE$^{+/-}$) at baseline.. Results expressed as mean ± SEM. CRE$^{+/-}$; *n*= 9, NOX5$^{+/-}$CRE$^{+/-}$; *n*= 9.

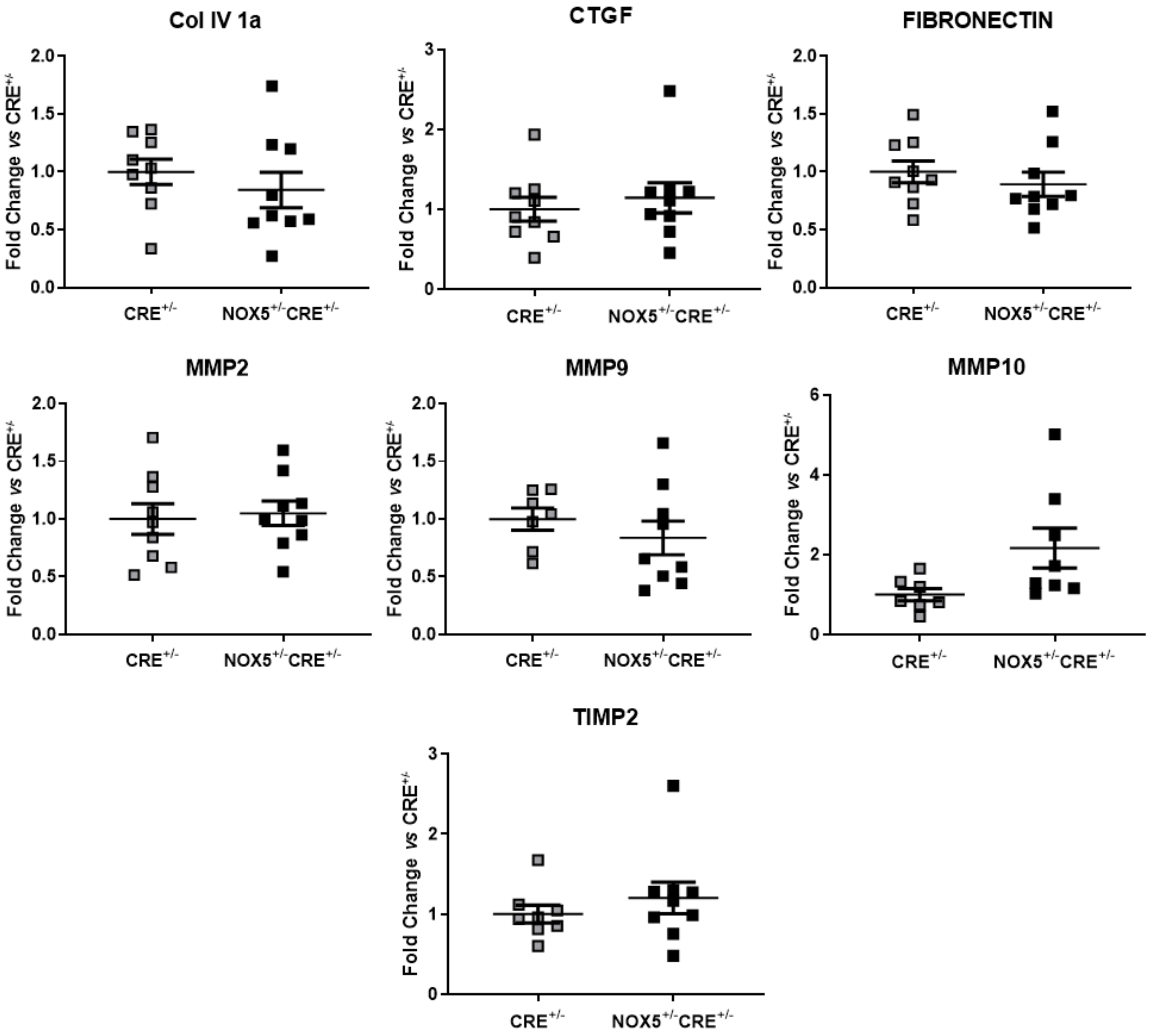

Fig. S2. mRNA expression of the components involved in cardiac fibrosis and extracellular matrix remodeling in the heart of healthy mice. Col IV 1a, CTGF, MMP2, MMP9, MMP10, TIMP2 and fibronectin mRNA expression in the NOX5-expressing mice (NOX5$^{+/-}$CRE$^{+/-}$) and the control mice (CRE$^{+/-}$) at baseline. Mann-Whitney test was used to analyze TIMP2 data. Results expressed as mean ± SEM. CRE$^{+/-}$; *n*= 9, NOX5$^{+/-}$CRE$^{+/-}$; *n*= 9.

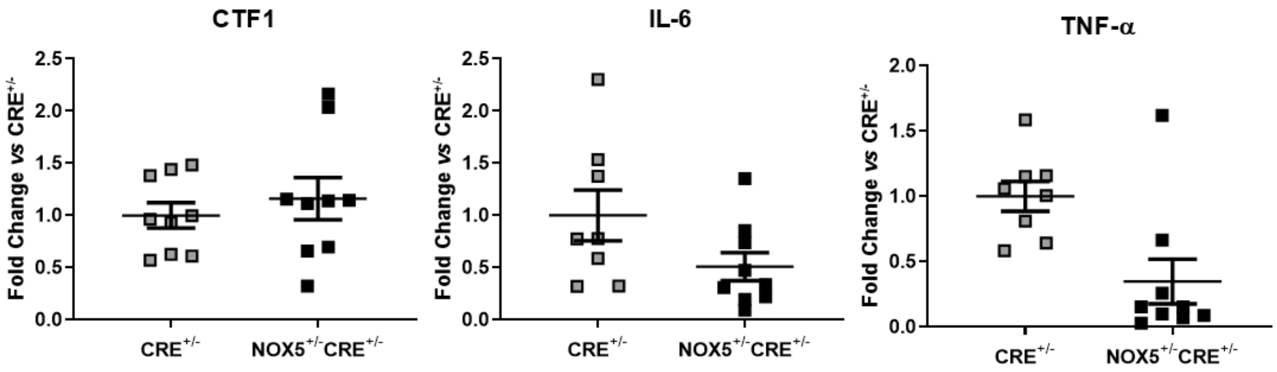

Fig. S3. mRNA expression of the inflammatory pathway components in the heart of healthy mice. CTF1, IL-6 and TNF-α mRNA expression in the NOX5-expressing mice (NOX5$^{+/-}$CRE$^{+/-}$) and the control mice (CRE$^{+/-}$) at baseline. Results expressed as mean ± SEM. CRE$^{+/-}$; *n*= 9, NOX5$^{+/-}$CRE$^{+/-}$; *n*= 9.

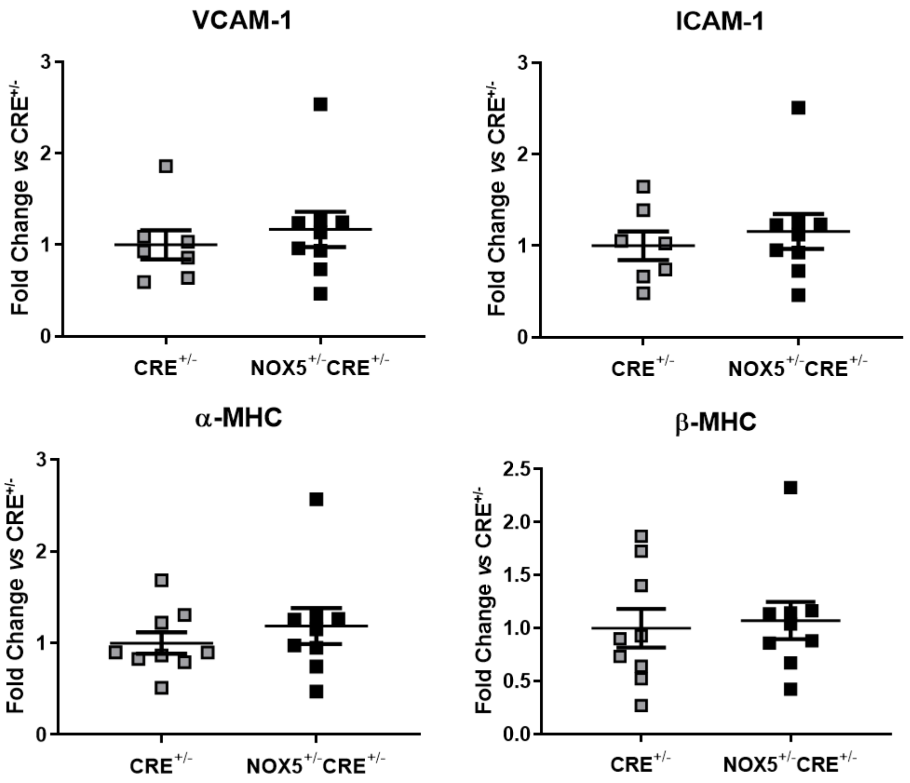

Fig. S4. mRNA expression of the adhesion molecules and myosin chains components in the heart of healthy mice. VCAM-1, ICAM-1, α-MHC and β-MHC mRNA expression in the NOX5-expressing mice (NOX5$^{+/-}$CRE$^{+/-}$) and the control mice (CRE$^{+/-}$) at baseline. *$p < 0.05$ *vs* control CRE$^{+/-}$ mice. . Mann-Whitney test was used to analyze VCAM-1, ICAM-1, α-MHC and β-MHC data. Results expressed as mean ± SEM. CRE$^{+/-}$; $n = 9$, NOX5$^{+/-}$CRE$^{+/-}$; $n = 9$.

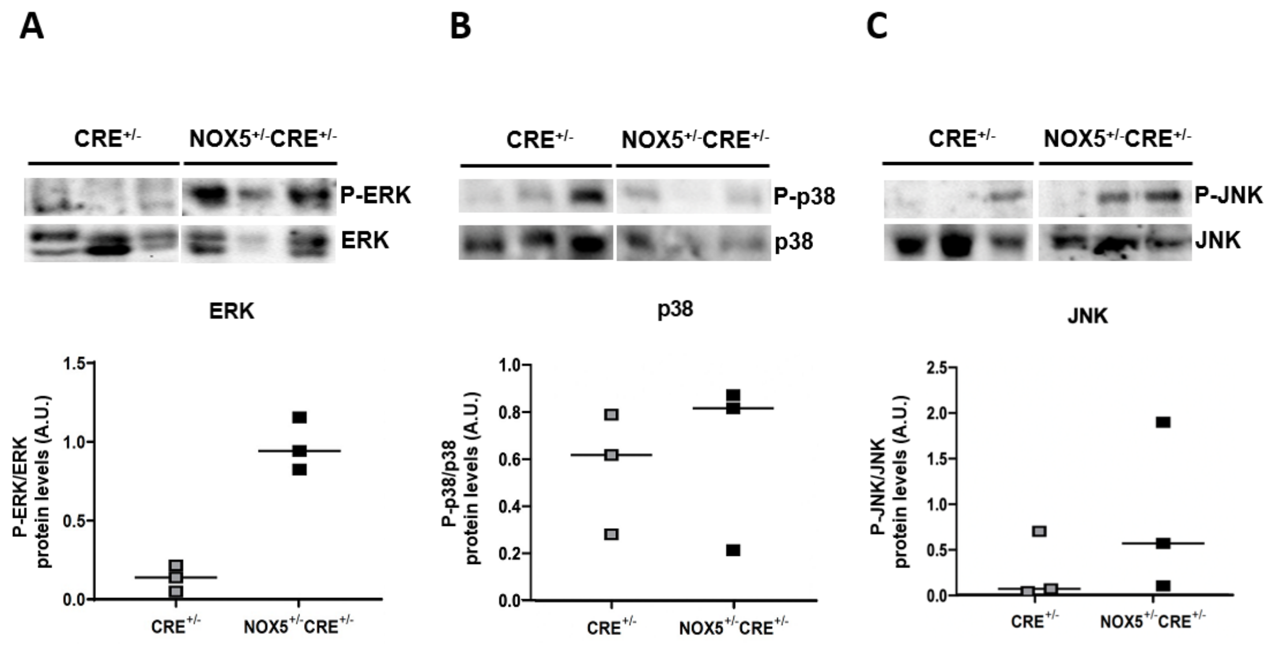

Fig. S5. MAPK family members phosphorylation in the heart of healthy mice. ERK, p38 and JNK protein phosphorylations in the control mice (CRE$^{+/-}$) and the NOX5-expressing mice (NOX5$^{+/-}$CRE$^{+/-}$) at baseline. Results expressed as individual values with median. CRE$^{+/-}$; *n*= 3, NOX5$^{+/-}$CRE$^{+/-}$; *n*= 3.

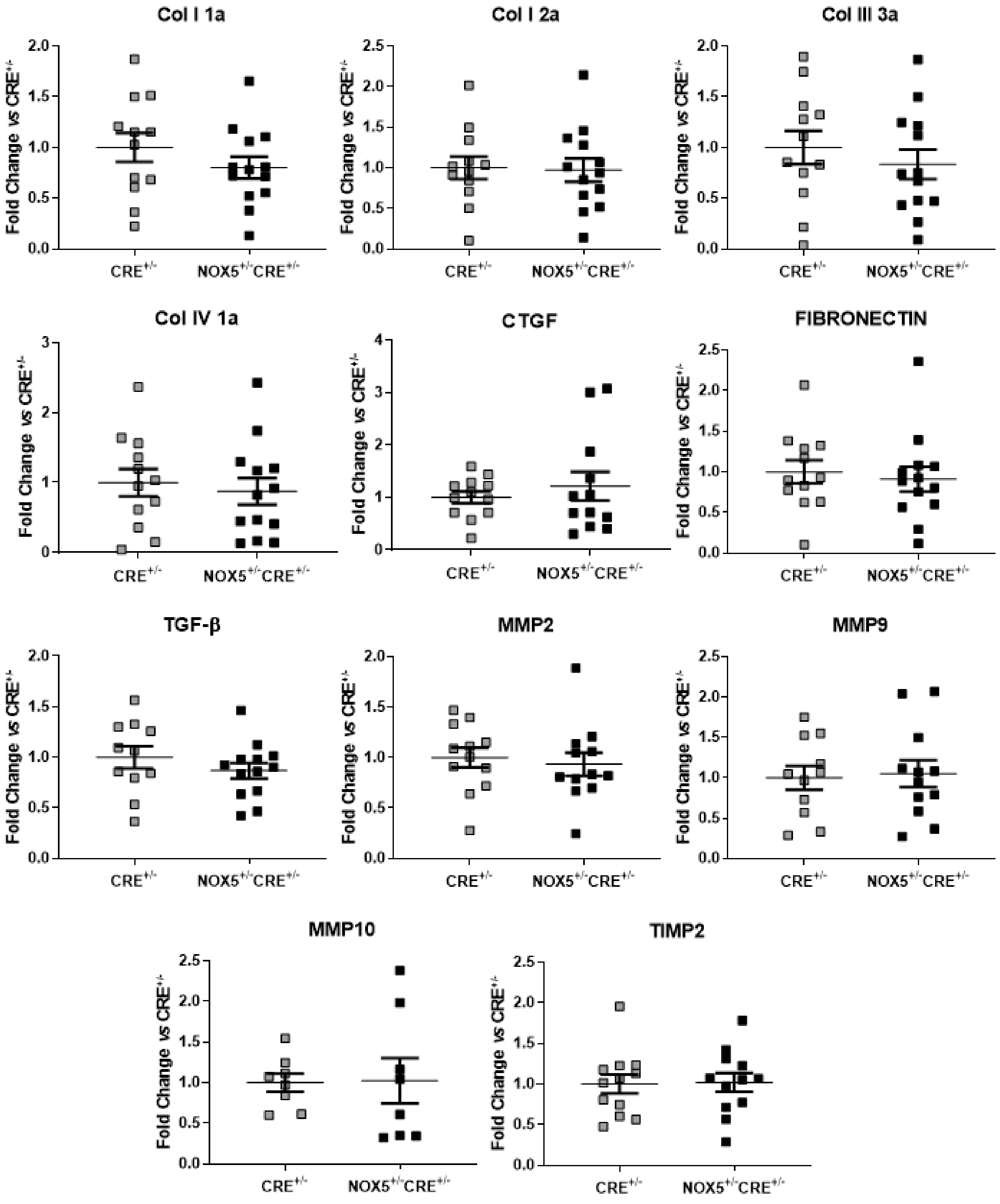

Fig. S6. mRNA expression of the components involved in cardiac fibrosis and extracellular matrix remmodeling in the heart of infarcted mice. Col I 1a, Col I 2a, Col III 1a, Col IV 1a, CTGF, Fibronectin, TGF-β,MMP2, MMP9, MMP10 and TIMP2 mRNA expression in the NOX5-expressing infarcted mice (NOX5$^{+/-}$CRE$^{+/-}$) and the control infarcted mice (CRE$^{+/-}$). Results expressed as mean ± SEM. CRE$^{+/-}$; *n*= 12, NOX5$^{+/-}$CRE$^{+/-}$; *n*= 13.

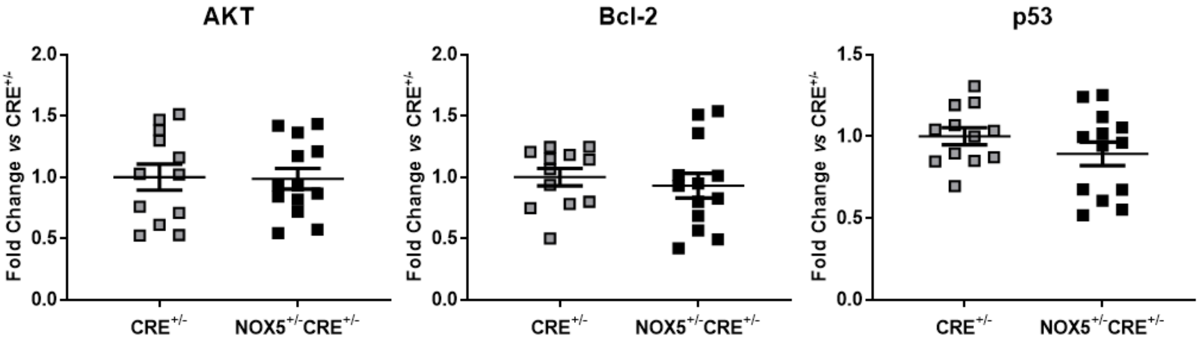

Fig. S7. mRNA expression of AKT, Bcl-2 and p53 in the heart of infarcted mice. AKT, Bcl-2 and p53 mRNA expression in the NOX5-expressing infarcted mice ($NOX5^{+/-}CRE^{+/-}$) and the control infarcted mice ($CRE^{+/-}$). Results expressed as mean ± SEM. $CRE^{+/-}$; *n*= 12, $NOX5^{+/-}CRE^{+/-}$; *n*= 13.

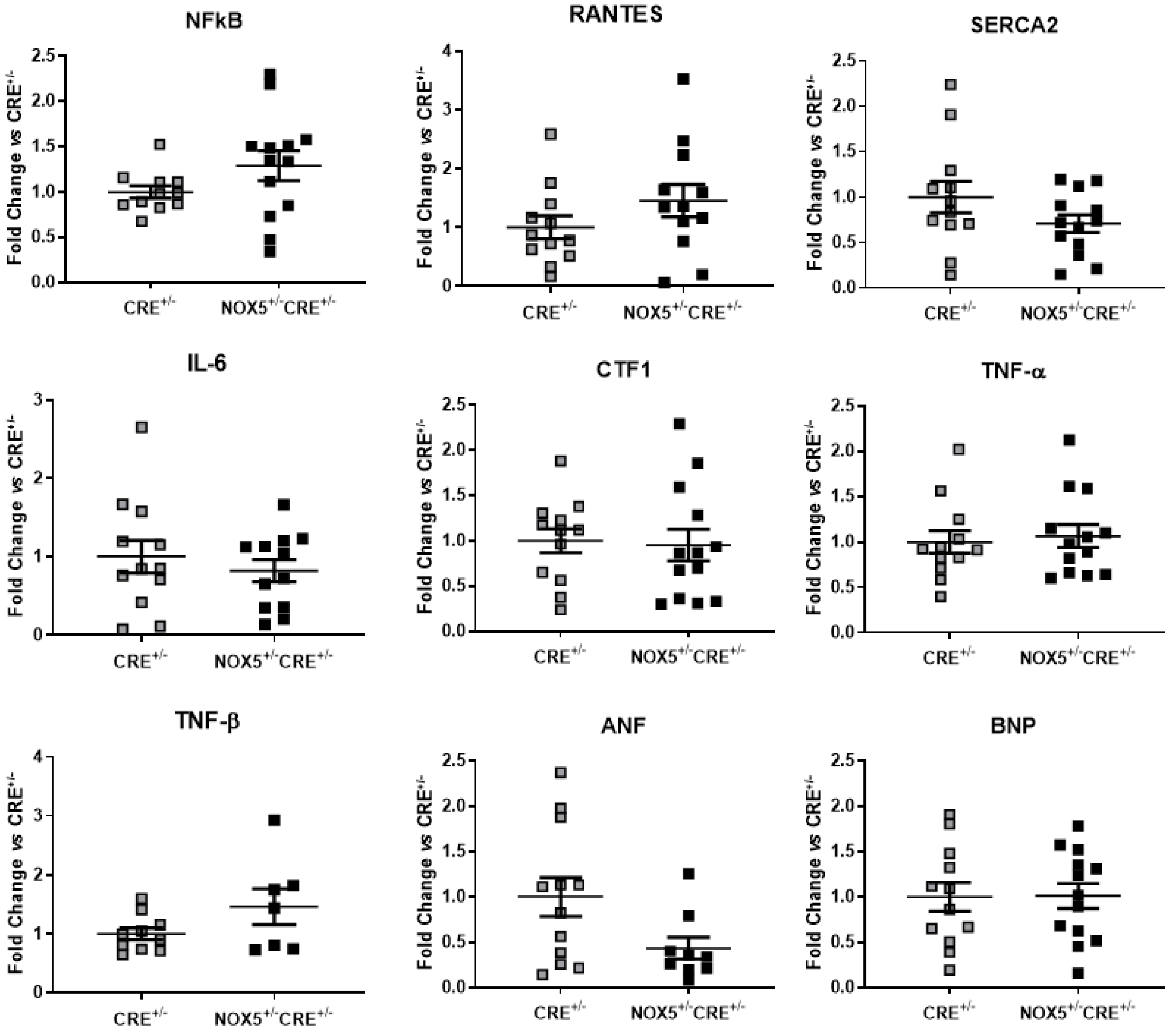

Fig. S8. mRNA expression of the inflammatory pathway components in the heart of infarcted mice. NFκB, RANTES, SERCA2, IL-6, CTF1, TNF-α, TNF-β, ANF and BNP mRNA expression in the NOX5-expressing infarcted mice ($NOX5^{+/-}CRE^{+/-}$) and the control infarcted mice ($CRE^{+/-}$). Mann-Whitney test was used to analyze ANF data. Results expressed as mean ± SEM. $CRE^{+/-}$; *n*= 12, $NOX5^{+/-}CRE^{+/-}$; *n*= 13.

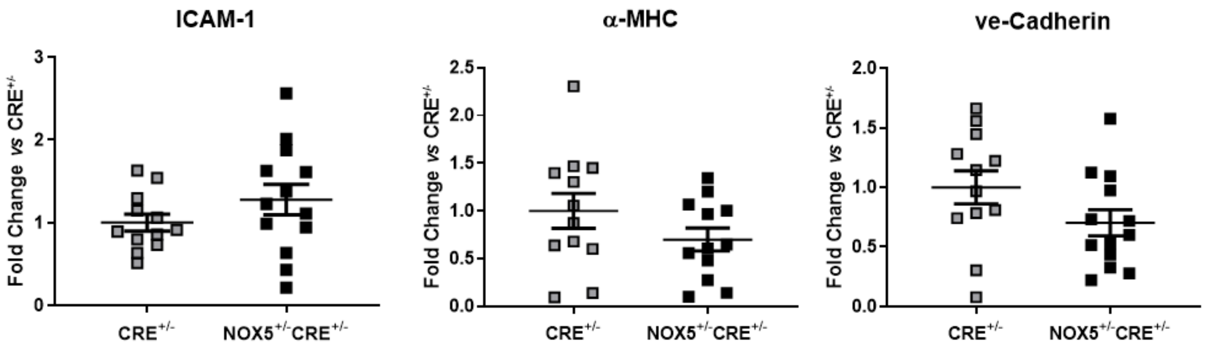

Fig. S9. mRNA expression of the adhesion molecules and myosin chains components in the heart of infarcted mice. ICAM-1, α-MHC and ve-cadherin mRNA expression in the NOX5-expressing infarcted mice ($NOX5^{+/-}CRE^{+/-}$) and the control infarcted mice ($CRE^{+/-}$). Results expressed as mean ± SEM. $CRE^{+/-}$; n= 12, $NOX5^{+/-}CRE^{+/-}$; n= 13.

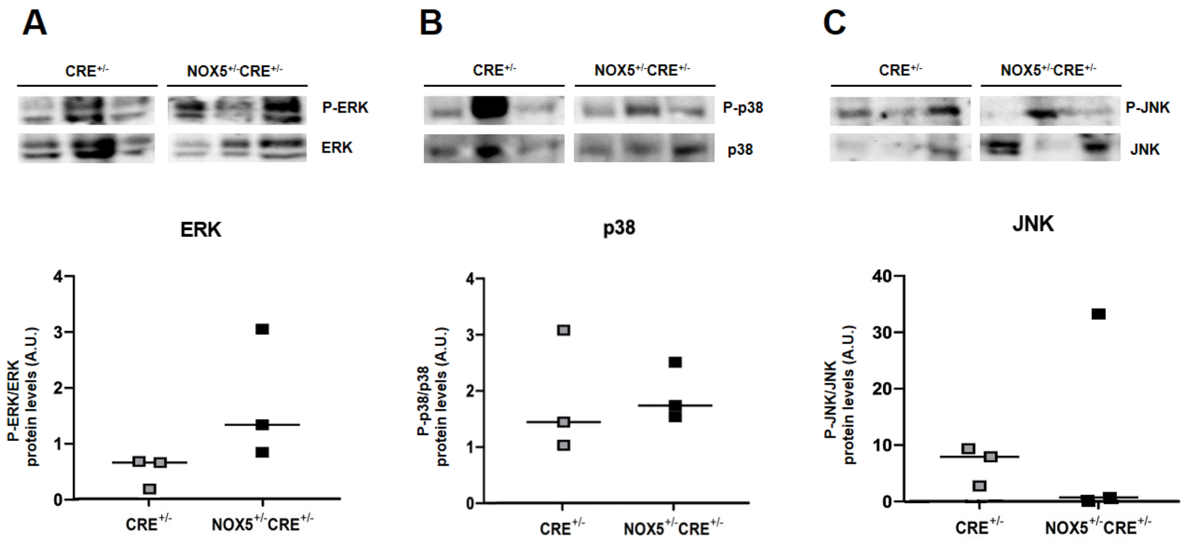

Fig. S10. MAPK family members phosphorylation in the heart of infarcted mice. ERK, p38 and JNK protein phosphorylations in the control mice (CRE$^{+/-}$) and the NOX5-expressing mice (NOX5$^{+/-}$CRE$^{+/-}$) at baseline. Results expressed as individual values with median. CRE$^{+/-}$; *n*= 3, NOX5$^{+/-}$CRE$^{+/-}$; *n*= 3.

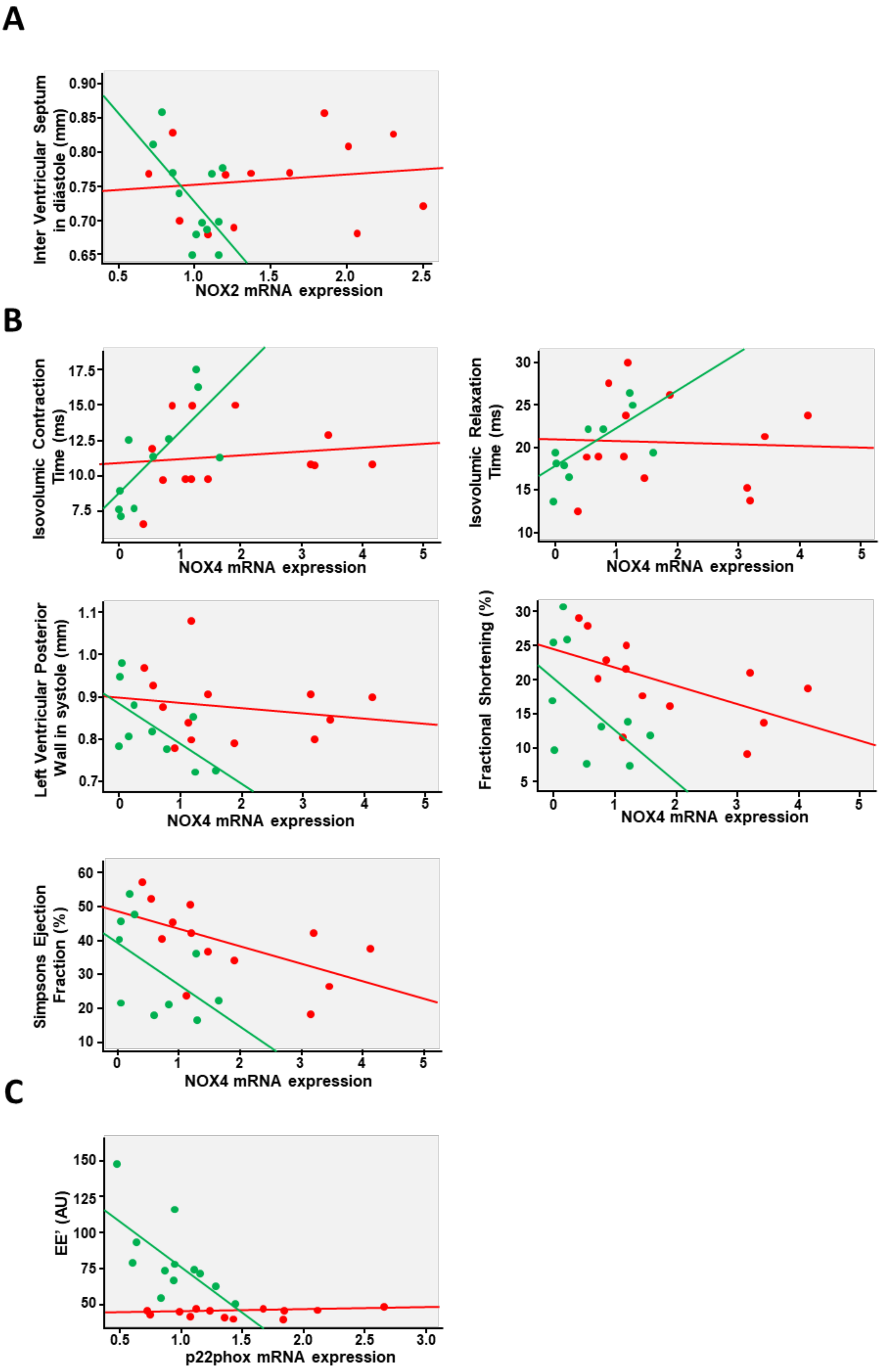

Fig. S11. Correlation analyses between echocardiographic parameters and the NOX components gene expression in $CRE^{+/-}$ (green) and $NOX5^{+/-}CRE^{+/-}$ (red) mice, after LAD ligation. (**A**) NOX2 with inter ventricular septum in diastole ($CRE^{+/-}$; R= -0.579, *p*= 0.049, $NOX5^{+/-}CRE^{+/-}$; R= 0.144, *p*= 0.640). (**B**) NOX4 with isovolumetric contraction time ($CRE^{+/-}$; R= 0.734, *p*= 0.016, $NOX5^{+/-}CRE^{+/-}$; R= 0.129, *p*= 0.674), isovolumetric relaxation time ($CRE^{+/-}$; R= 0.687, *p*= 0.028, $NOX5^{+/-}CRE^{+/-}$; R= -0.046, *p*= 0.882), left ventricular posterior wall in systole ($CRE^{+/-}$; R= -0.649, *p*= 0.042, $NOX5^{+/-}CRE^{+/-}$; R= -0.187, *p*= 0.541), fractional shortening ($CRE^{+/-}$; R= -0.537, *p*= 0.110, $NOX5^{+/-}CRE^{+/-}$; R= -0.558, *p*= 0.047) and Simpson ejection fraction ($CRE^{+/-}$; R= -0.543, *p*= 0.105, $NOX5^{+/-}CRE^{+/-}$; R= -0.562, *p*= 0.046). (**C**) p22phox with EE' ($CRE^{+/-}$; R= -0.646, *p*= 0.023, $NOX5^{+/-}CRE^{+/-}$; R= 0.260, *p*= 0.392). AU, arbitrary units. $CRE^{+/-}$; *n*= 12, $NOX5^{+/-}CRE^{+/-}$; *n*= 13.

A

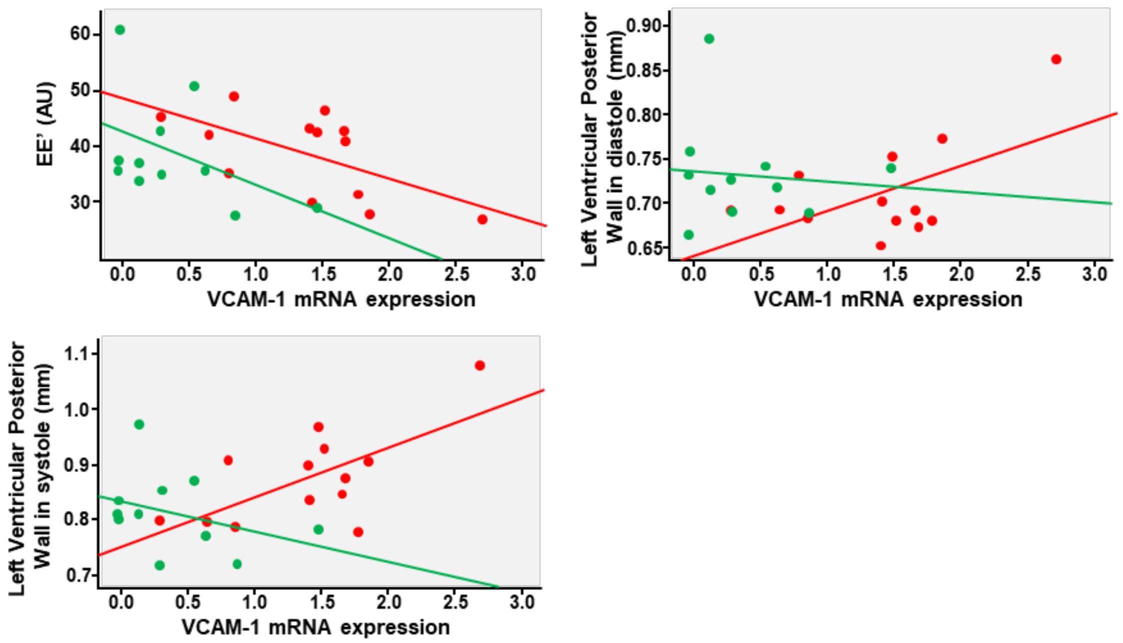

B

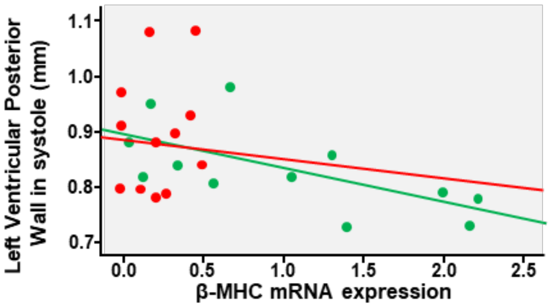

Fig. S12. Correlation analyses between echocardiographic parameters and V-CAM1 or β-MHC gene expression in CRE$^{+/-}$ and NOX5$^{+/-}$CRE$^{+/-}$ mice, after LAD ligation. (**A**) VCAM-1 with (CRE$^{+/-}$; R= -0.452, *p*= 0.163, NOX5$^{+/-}$CRE$^{+/-}$; R= -0.597, *p*= 0.031), left ventricular posterior wall in diastole (CRE$^{+/-}$; R= -0.099, *p*= 0.771, NOX5$^{+/-}$CRE$^{+/-}$; R= 0.565, *p*= 0.044) and left ventricullar posterior wall in systole (CRE$^{+/-}$; R= -0.351, *p*= 0.291, NOX5$^{+/-}$CRE$^{+/-}$; R= 0.663, *p*= 0.013). (**B**) β-MHC with left ventricular posterior wall in systole (CRE$^{+/-}$; R= -0.637, *p*= 0.026, NOX5$^{+/-}$CRE$^{+/-}$; R= -0.077, *p*= 0.823). AU, arbitrary units. CRE$^{+/-}$; *n*= 12, NOX5$^{+/-}$CRE$^{+/-}$; *n*= 13.

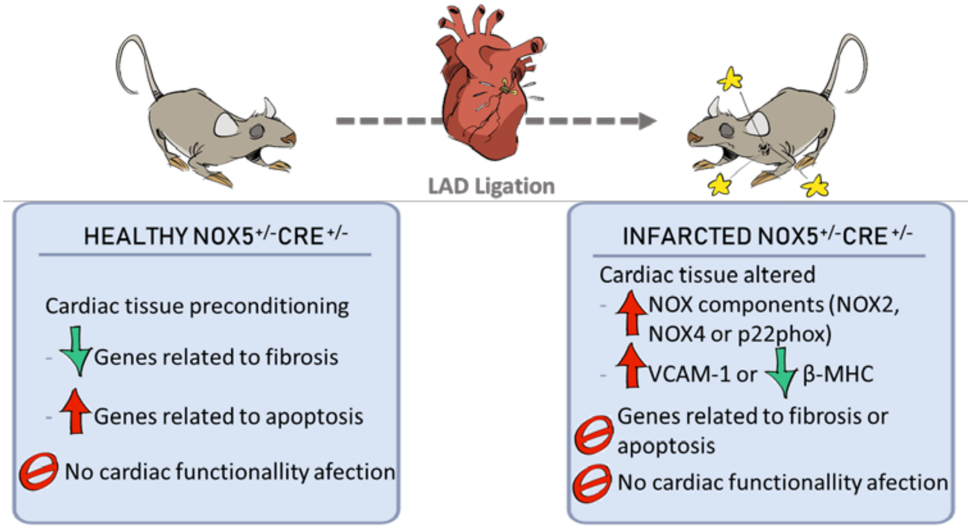

Fig. S13. Summary illustration
